# Effects of Polymer Matrices and Carbon Nanotubes on the Generation of Electric Energy in a Microbial Fuel Cell

**DOI:** 10.3390/membranes8040099

**Published:** 2018-10-25

**Authors:** Yulia Plekhanova, Sergei Tarasov, Vladimir Kolesov, Iren Kuznetsova, Maria Signore, Fabio Quaranta, Anatoly Reshetilov

**Affiliations:** 1 FSBIS G.K. Skryabin Institute of Biochemistry and Physiology of Microorganisms, Russian Academy of Sciences, Pushchino 142290, Russia; plekhanova@ibpm.pushchino.ru (Y.P.); setar25@gmail.com (S.T.); 2 FSBIS V.A. Kotelnikov Institute of Radio Engineering and Electronics, Russian Academy of Sciences, Moscow 125009, Russia; kvv@cplire.ru (V.K.); kuziren@yandex.ru (I.K.); 3 CNR, Institute for Microelectronics and Microsystems, Via Monteroni, 73100 Lecce, Italy; mariaassunta.signore@cnr.it (M.S.); fabio.quaranta@cnr.it (F.Q.)

**Keywords:** microbial fuel cell, polymer matrix, immobilization of bacterial cells, interaction of cell membranes with carbon nanotubes, boost converter accumulation

## Abstract

The anode of a microbial fuel cell (MFC) was formed on a graphite electrode and immobilized *Gluconobacter oxydans* VKM-1280 bacterial cells. Immobilization was performed in chitosan, poly(vinyl alcohol) or *N*-vinylpyrrolidone-modified poly(vinyl alcohol). Ethanol was used as substrate. The anode was modified using multiwalled carbon nanotubes. The aim of the modification was to create a conductive network between cell lipid membranes, containing exposed pyrroloquinoline quinone (PQQ)-dependent alcoholdehydrogenases, and the electrode to facilitate electron transfer in the system. The bioelectrochemical characteristics of modified anodes at various cell/polymer ratios were assessed via current density, power density, polarization curves and impedance spectres. Microbial fuel cells based on chitosan at a matrix/cell volume ratio of 5:1 produced maximal power characteristics of the system (8.3 μW/cm^2^) at a minimal resistance (1111 Ohm cm^2^). Modification of the anode by multiwalled carbon nanotubes (MWCNT) led to a slight decrease of internal resistance (down to 1078 Ohm cm^2^) and to an increase of generated power density up to 10.6 μW/cm^2^. We explored the possibility of accumulating electric energy from an MFC on a 6800-μF capacitor via a boost converter. Generated voltage was increased from 0.3 V up to 3.2 V. Accumulated energy was used to power a Clark-type biosensor and a Bluetooth transmitter with three sensors, a miniature electric motor and a light-emitting diode.

## 1. Introduction

Efficiency of microbial fuel cells (MFCs) directly depends on the efficiency and sustainability of biocatalyst operation. In most MFCs developed to date, cells are attached to the electrode surface by adsorption. Adsorption is a simple and efficient immobilization technique not affecting the physiological properties of microorganisms, but its drawbacks are insecure attachment of biocatalysts and poor protection from external factors. Entrapment of microorganisms in polymer gels contributes to the preservation of their physiological activity, stimulates metabolism, and protects cells from adverse agents [1,2,3,4].

Polymers used to immobilize biocatalysts on conductive electrodes can be divided into two groups—natural and synthetic. Natural polymers include polysaccharides, alginic acid salts, carrageenans, chitin and chitosan, gelatin, agar, etc., as well as protein hydrogels. Synthetic matrices for immobilization comprise poly(vinyl alcohol), polyacrylamide, and polyethylene glycol. Smart polymers, subject to strong conformational changes at minor changes in the environment, can be both natural and synthetic. Polymer matrices (e.g., polyacetylene, polypyrrol, polyaniline, etc., in which carbon, graphite, nanoparticles of silver and other metals are used as conductive components) are also used. Various combinations of polymers with nanostructures, such as nanoparticles, nanotubes and nanoengineered smart polymers, enable matrices with novel properties providing for higher stability, sensitivity, biocompatibility, etc. [5].

Among natural polymer gels, chitosan gel is one of the most widespread agents to immobilize microbial cells [6,7]. Immobilization in chitosan gel is used in MFCs because chitosan, due to its large pores, enables the development of biofilms on the electrode without preventing the flow of nutrient substrates to cells [8]. Besides, carbon nanotube/chitosan nanocomposites are used to increase the power of microbial fuel cells [9,10]. On the whole, the use of chitosan, owing to its mechanical strength and structure, has a positive effect on operational stability and analytical characteristics of MFCs [11].

Advantages of synthetic polymers include the possibility of developing carriers with preset properties. Gels based on poly(vinyl alcohol) (PVA) possess a high micro- and macroporosity, which ensures the mass transfer of substrates. Moreover, they exhibit great thermal stability, high resistance to biological degradation and, in practice, insensitivity to the composition of the medium (dissolved substances, buffer, pH) [12]. Gel based on poly(vinyl alcohol) is a biologically compatible [13], nontoxic [14], readily available and cheap polymer. In reference [15], PVA-based gel has been used for immobilization of *Photobacterium phosphoreum* to study effects of various toxic substances (phenol, pentachlorophenol, some metal ions, 2,4-dichlorophenoxyacetic acid and 2,4,5-trichlorophenoxyacetic acid) on bacterial luminescence. It has been shown to ensure a long-time stability and intensity of luminescence in biosensor operation. Modification of PVA by, e.g., *N*-vinylpyrrolidone (mPVA) enables a more stable operation of biosensor receptor elements and MFCs [16,17]. *N*-vinylpyrrolidone not only is nontoxic but also enhances the activity of the enzyme systems in some microorganisms [18].

The high resistance of polymer gels necessitates the use of additional materials in bioelectrode constructions, e.g., carbon nanotubes. In reference [19], the authors describe a mechanism according to which covalent binding forces can arise between lipid membranes and carbon nanotubes. In reference [20], an assumption is hypothetically considered that, as the distance between the enzyme’s active centre and the graphite electrode decreases, at a critical distance between them conditions can arise for a facilitated transfer of charge from the electrode to the enzyme. The same interaction pattern, not necessarily leading to mediator-free transfer but facilitating charge transfer, can be assumed to exist for whole bacterial cells, where PQQ (pyrroloquinoline quinone)-dependent dehydrogenases incorporated into the lipid envelope of cell membranes will be an analogue of the enzyme.

*Gluconobacter oxydans* are frequently used for biotechnological applications, because these bacteria have high growth rates when cultivated on growth media, possess a high metabolic activity, are relatively stable in immobilization and require no external cofactor for their PQQ-dependent dehydrogenases to function [21,22,23]. Besides, they have a significant number of membrane-localized enzyme complexes (aldose and alcohol dehydrogenases capable of oxidizing quite a number of carbohydrates and alcohols), which simplifies electron transfer in the system and enables their use in biosensors and as an MFC biocatalyst [22,24,25,26,27,28,29]. In adsorption contact of bacterial cells with carbon nanotubes, a quite possible situation is, according to the principle of random interaction, to form covalent bonds between lipids, that enclose PQQ-dependent dehydrogenases, and nanotubes. Due to these properties, *Gluconobacter* cells are ideal for test studies.

The aim of the work was to assess the electrochemical characteristics of MFCs based on *G. oxydans* bacterial cells immobilized into the following polymers: chitosan, poly(vinyl alcohol) and *N*-vinylpyrrolidone-modified poly(vinyl alcohol). The developed MFCs were modified by multiwalled carbon nanotubes (MWCNT) and used to accumulate electric energy via a boost converter to power a biosensor electrode, Bluetooth transmitter, miniature electric motor and light-emitting diode.

## 2. Materials and Methods

### 2.1. Reagents

We used dibasic trihydrate potassium phosphate, sodium hydroxide, sodium chloride, ethanol, acetic acid (ZAO Mosreaktiv, Moscow, Russia); 2,6-dichlorophenolindophenol sodium salt; low molecular weight chitosan; Mowiol [poly(vinyl alcohol) (M = 115,000 amu)]; *N*-vinylpyrrolidone; ammonium cerium nitrate (Sigma-Aldrich, St. Louis, MO, USA); sorbitol, yeast extract, and Bacto-agar (Difco, Detroit, MI, USA). Graphite electrodes (S-3M, OOO Poliprof-L, Moscow, Russia) were used as working electrodes. Taunit-M MWCNT (OOO NanoTekhCentre, Tambov, Russia) were applied to modify anodes.

Strain *Gluconobacter oxydans* sbsp. *industrius* VKM-1280 (All-Russian Collection of Microorganisms) was used. Cells were cultivated as described in reference [30].

Gel based on poly(vinyl alcohol) was modified by *N*-vinylpyrrolidone according to the method presented in [31] by V.A. Arlyapov and V.A. Alferov (Tula State University, Tula, Russia). For this, 0.2–0.8 mL of an aqueous solution of ammonium cerium nitrate and 0.3–0.1 mL of *N*-vinylpyrrolidone were added to 20 mL of a 5% aqueous solution of PVA at constant mixing. The mixing was done at 40 °C in a nitrogen atmosphere for 3 h.

### 2.2. Formation of the Working Electrode

Graphite rods (2 mm in height and 3 mm in radius) were used as a base material for the anode and cathode. Graphite rods (65 mm in height and 3 mm in radius) of total area 7.5 cm^2^ were used as a base material for converter accumulation of energy.

A biocatalyst, a mixture of a bacterial cell suspension (1 mg wet weight/μL) and polymer (chitosan, a 2% solution in 1% acetic acid [32]; PVA or mPVA) at various volume ratios, was used. The produced mixture was applied onto the electrode and dried at room temperature; the concentration of cells on the electrode surface was 0.12 mg wet weight/mm^2^. To introduce nanomaterial into the electrode composition, 0.4 mg MWCNT powder was mixed with a 20-μL suspension of bacterial cells; the mixture was left at a temperature of 4 °C for 60 min. Then, a 10-μL polymer solution was added. The produced solution was applied onto the electrode and dried at a temperature of 22 °C. The contents of cells and nanomaterial on the electrode surface were 0.12 mg wet weight/mm^2^ and 1.57 μg/mm^2^, respectively.

### 2.3. MFC Setup and Operation

**Figure 1:**
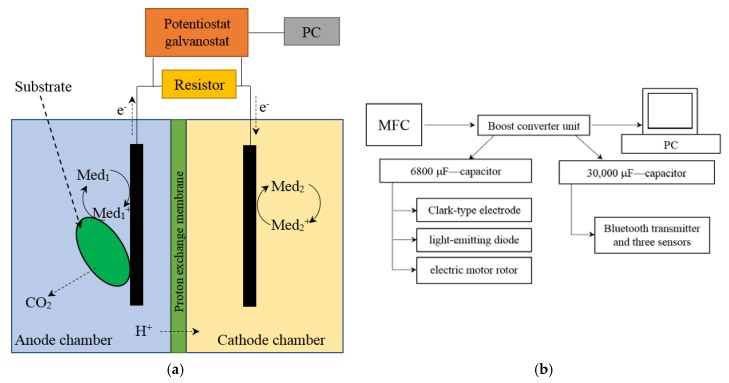
Scheme for the MFC and its external connections (**a**); block diagram for converter accumulation of energy (**b**).

The microbial fuel cell was represented by two interconnected cuvettes; the volumes of the anode and cathode chambers were 5 mL. The chambers were separated by an MF-4SK proton-selective membrane (Plastpolimer, St.-Petersburg, Russia), an analogue of the Nafion 117 membrane in a protonated form. The area of the membrane was 1.2 cm^2^. Interconnected cuvettes, in which the volumes of the anode and cathode chambers were equal to 50 mL, were used for converter accumulation of energy. The area of the proton-selective membrane was 20 cm^2^. Scheme for the MFC and its external connections is presented in Figure 1a. Block diagram for converter accumulation of energy is presented in Figure 1b.

A 25 mM potassium phosphate buffer, pH 6.5, containing 10 mM sodium chloride, was used as a background solution; as mediators, we used 2,6-dichlorophenolindophenol (DCPIP, 42 μM) in the anode chamber and potassium hexacyanoferrate (III) (HCF, 4 mM) in the cathode chamber. Ethanol at a concentration of 10 mM was used as substrate for *G. oxydans* cells. Cyclic voltammograms were registered at a scan rate of 3 mV/s. Impedance measurements were carried out at applied potentials of 0 V and 200 mV within the frequency range of 40 kHz to 0.02 Hz with an AC signal of a 10-mV amplitude. The measurements were conducted at a constant stirring of solutions.

The current–voltage and impedance characteristics were measured on a VersaSTAT 4 potentiostat galvanostat (Ametek Inc., Berwyn, PA, USA). The anode of the MFC was used as the working electrode and the cathode was used as the reference as well as the counter electrode.

The MFC power characteristics and its internal resistances were calculated by the formulas from reference [33]. The values of *R*_in_ and *P* were normalized for the electrode surface area and were used to compare the characteristics of modified electrodes.

## 3. Results and Discussion

### 3.1. Applied Potential Selection

**Figure 2:**
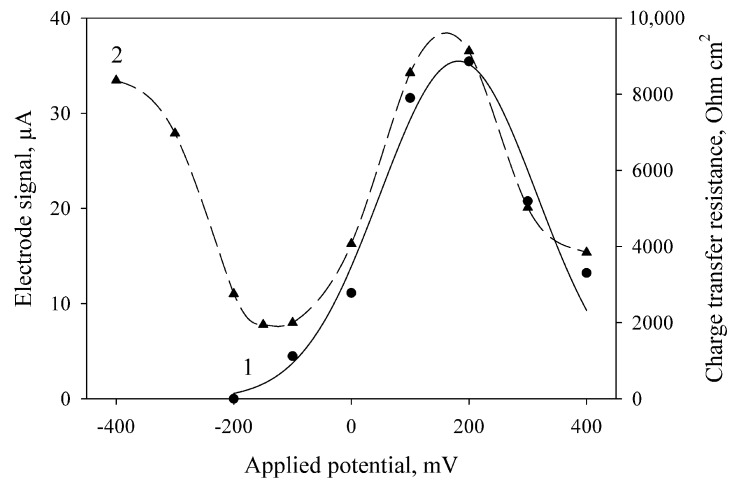
Effect of the applied potential on the electrode signals at the introduction of ethanol (1) and on the electrode charge transfer resistance measured by electrochemical impedance spectroscopy after addition of ethanol (2). The electrode was modified using bacterial cells and chitosan (2:1, *v*/*v*).

The amplitude of current generated by the MFC at the oxidation of ethanol by bacterial cells depends on the applied potential (Figure 2, curve 1). The maximal generated current of the electrode in the presence of mediator was observed at an applied potential of 200 mV. These results correlate with the literature data (the standard redox potential of DCPIP mediator is +0.217 V [34]). Also, we obtained the values of charge transfer resistance (*R*_ct_) at various applied potentials by electrochemical impedance spectroscopy (Figure 2, curve 2). The minimal values of *R*_ct_ were obtained at an applied potential of −150 to −180 mV; maximal, at 200 mV. These dependences were also obtained for MFCs with bacterial cells immobilized into PVA and mPVA (data not shown). For this reason, further amperometric measurements were carried out at an applied potential of 200 mV, and the impedance measurements were conducted at an applied potential of −150 mV.

### 3.2. Behaviour of Bacterial Cells in Polymer Gels

**Figure 3:**
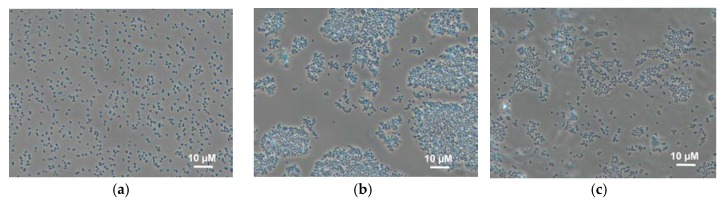
Micrographs of *Gluconobacter oxydans* cells in polymer gels: (**a**) chitosan; (**b**) PVA; (**c**) mPVA.

The effect of the immobilization matrix on cells was investigated by light microscopy. Cells were mixed with various polymers (Figure 3) under the same conditions and their behaviour was compared. The micrographs show that cells mixed with chitosan are uniformly distributed along the matrix, and this state does not change with time. In PVA gel, cells form conglomerates immediately after mixing; the size of the conglomerates changes with time. This can be due to the unfavourable impact of this matrix on bacterial cells, as the result of which bacteria tend to minimize the contact with the matrix. This effect is also observed for *N*-vinylpyrrolidone-modified PVA; however, conglomerates are smaller. This can be due to a decrease of agglomeration of PVA in the case of its modification by various compounds similar to those described in references [14,35].

The presented data suggest that, when using PVA and mPVA, only those cells that are on the outer boundary of conglomerates are in contact with the electrode surface. This means that the total electric signal from the same number of cells should be theoretically higher in the case of using chitosan gel.

### 3.3. Effect of the Concentration of Polymer and Bacterial Cells on the Electrochemical Characteristics of MFC Bioanode

**Figure 4:**
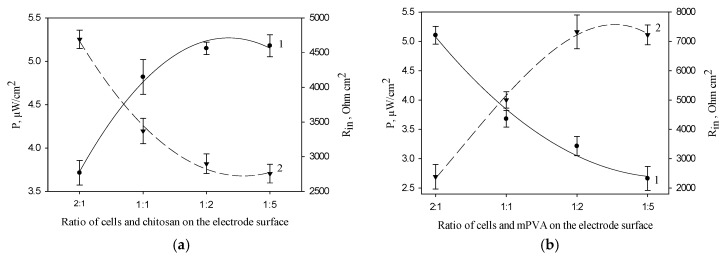
Effect of the ratio of cells and polymer on the anode surface on MFC power (1) and internal resistance (2) (the number of cells on the electrode surface is constant, 0.12 mg/mm^2^): 1, MFC power; 2, *R*_in_; (**a**) chitosan; (**b**) mPVA.

Figure 4a shows the dependences of MFC power and internal resistance on the ratio of cells and chitosan polymer on the anode surface. The maximal values of MFC power and the minimal values of MFC internal resistance were obtained at a cell/chitosan volume ratio of 1:5. Figure 4b presents the same characteristics for the electrode based on mPVA polymer. The maximal values of MFC power and the minimal values of MFC internal resistance were obtained at a cell/mPVA volume ratio of 2:1. A similar dependence was also obtained for PVA polymer (data not shown). The obtained data for synthetic polymers can be due to the self-organization of Gluconobacter cells in the PVA and mPVA matrices (Figure 3), the extent of which increases at a prolonged contact with polymer [36] and, possibly, at an increase of its amount.

We obtained dependences of MFC power and internal resistance for these polymers at various concentrations of bacterial cells on the anode surface mixed at ratios of 1:5 (cells/chitosan, *v*/*v*) and 2:1 (cells/PVA or mPVA, *v*/*v*). Variation of the concentration of cells on the anode surface within the range of 0.012–0.35 mg/mm^2^ for all polymers had no significant effect on the electrochemical parameters of the MFCs.

**Figure 5:**
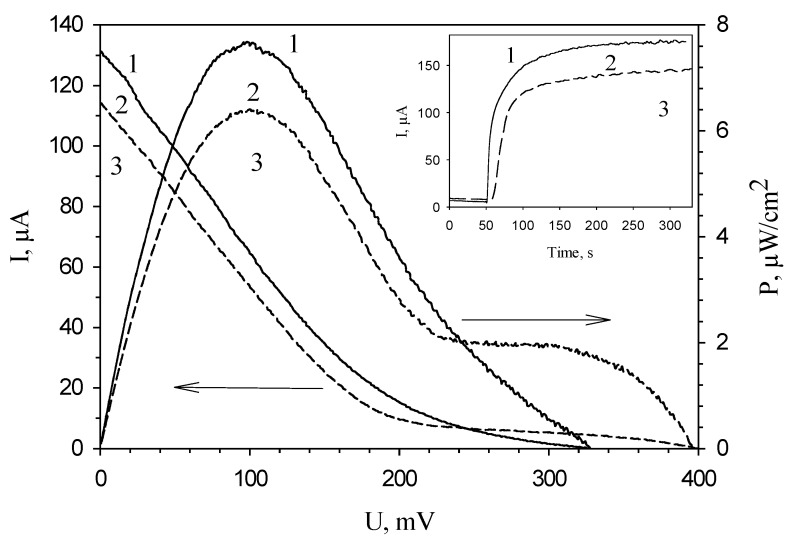
Typical polarization curves obtained from cyclic voltammograms and, calculated based on them, power characteristics of polymer/*G. oxydans* electrodes: 1, chitosan; 2, mPVA; 3, PVA. Inset: typical signals of polymer/*G. oxydans* electrodes: 1, chitosan; 2, mPVA; 3, PVA.

Inset of Figure 5 shows typical MFC signals in response to the introduction of ethanol at an applied potential of 200 mV. The maximal amplitude of the signal was observed for an MFC in which chitosan was used as an immobilization agent. Figure 4 presents typical polarization curves for an MFC modified by various polymers, and MFC power characteristics calculated from them. Correspondingly, the minimal values of MFC internal resistance and the maximal values of developed power are also observed for an MFC modified by chitosan (Table 1). The table compares the characteristics of the main bioelectrochemical parameters for MFCs modified by various polymers as measured by voltammetry and impedance spectroscopy. All values of resistances obtained by impedance spectroscopy were measured at an applied potential of −150 mV, because at this potential the MFC internal resistance is minimal, as seen in Figure 2, curve 2. Table 1 presents both the electrode charge transfer resistances for three types of bioanodes (*R*_ct_) and the total internal resistances of the systems (*R*_in_). The value of anode resistance is up to 75% of the total internal resistance of the system, which once again emphasizes the importance of modifying bioanodes with the view of reducing their resistances. The contribution of chitosan to the bioanode resistance is the least of the three investigated polymers; therefore, chitosan ensures a reliable attachment of material and does not prevent vital activities of cells. Thus, the use of chitosan for immobilization of microorganisms makes it possible to achieve the highest MFC power.

### 3.4. Modification of the Anode by Carbon Nanotubes

To improve the conductance and increase the active area of the surface, the bioanode is modified by various nanomaterials, including nanotubes [10,37,38]. In this work we used multiwalled carbon nanotubes, because earlier they have been shown to be less toxic for bacterial cells than single-walled nanotubes [39].

Cells of *Gluconobacter oxydans* differ from many other bacteria by the arrangement of their enzyme complexes [26]. Pyrroloquinoline quinone-dependent dehydrogenases are exposed to the outer side of the cell lipid membrane, which simplifies the access of substrates to them. Modification of the bioanodes by carbon nanotubes may create conductive channels constructed from nanotubes that are distinguished by minimal resistance. Nanotubes, being in immediate contact with cell lipid membranes provide for facilitated transfer of electrons from enzyme complexes of bacteria to the surface of graphite electrodes via the layer of the immobilization matrix. Such a network should theoretically reduce the bioanode impedance and MFC resistance, which would ultimately increase the generated power of the MFC [40].

**Figure 6:**
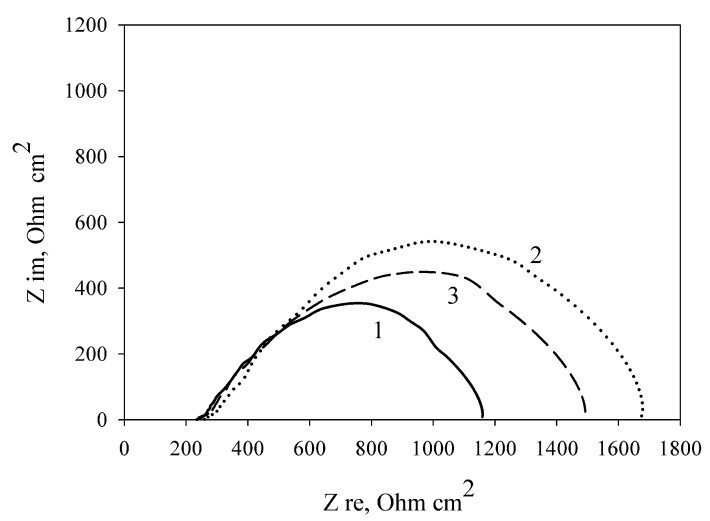
Nyquist plots of the impedance spectra of MWCNT-modified polymer/*G. oxydans* electrodes: 1, chitosan; 2, mPVA; 3, PVA.

Table 2 shows the MFC characteristics at the modification of the electrode by carbon nanotubes immobilized with cells in three polymers. As seen from Table 1 and Table 2, modification of a PVA/*G. oxydans* electrode by carbon nanotubes increases the MFC power by 20.7% (from 6.56 to 7.92 μW/cm^2^); thereby, the internal resistance decreases by only 2.5% (from 1251 to 1219 Ohm cm^2^). Modification of an mPVA/*G. oxydans* electrode by carbon nanotubes also led to a rise in the MFC power by 18.5% (from 6.10 to 7.23 μW/cm^2^) and a decrease of the internal resistance by 12.2% (from 1455 to 1277 Ohm cm^2^). Modification of a chitosan/*G. oxydans* electrode by nanotubes caused a power increase of 38.4% (from 7.63 to 10.56 μW/cm^2^) and a decrease of internal resistance by 12.1% (from 1111 to 977 Ohm cm^2^). Figure 6 shows the impedance spectres of MWCNT-modified electrodes depending on the used polymer. The lowest charge transfer resistance was demonstrated by an electrode with bacterial cells immobilized using chitosan. Thus, modification of an MFC anode by carbon nanotubes leads to an increase of MFC power and a decrease of internal resistance as compared with the nonmodified anode when using any of the three investigated polymers.

### 3.5. Applications of MWCNT/Chitosan/G. oxydans MFC

Microbial fuel cells are low-power supply sources (10^−7^–10^−3^ W/cm^2^). Still, MFC applications are limited by not only their low power but also the low value of generated voltage. In this context, a topical problem for their broader application is to increase the MFC output voltage. To increase MFC energy by increasing voltage, this work used a boost converter based on a bq 25504 integrated circuit that performs direct current transformation [41]. In the general case, the converter accumulates and stores electric energy generated by unstable sources. As applied to MFCs, the method of increasing voltage by means of a converter has not been reported earlier.

We developed a system connecting a bq 25504-based converter to an MFC [42] and worked out various modes of increasing and storing output voltage, which in practice corresponded to accumulation of electric energy. This system increased MFC voltage from open-circuit potential (0.3 V) to a preset value of 3.2 V. The time of accumulating the preset voltage across a 6800-μF capacitor depends on the number of MFCs connected to the converter and their modifications (Table 3).

It is seen from the data presented that in a single MFC the generated voltage is too low for an efficient start of the converter and capacitor charge. This charging feature is due to the characteristic of the used integrated circuit bq 25504, which requires an input voltage of 300 mV and higher. Series connection of two MFCs makes it possible to sum up their voltages to ensure the converter start voltage. Therefore, the internal resistance of the system does not decrease, but the total voltage output of the MFC increases.

**Figure 7:**
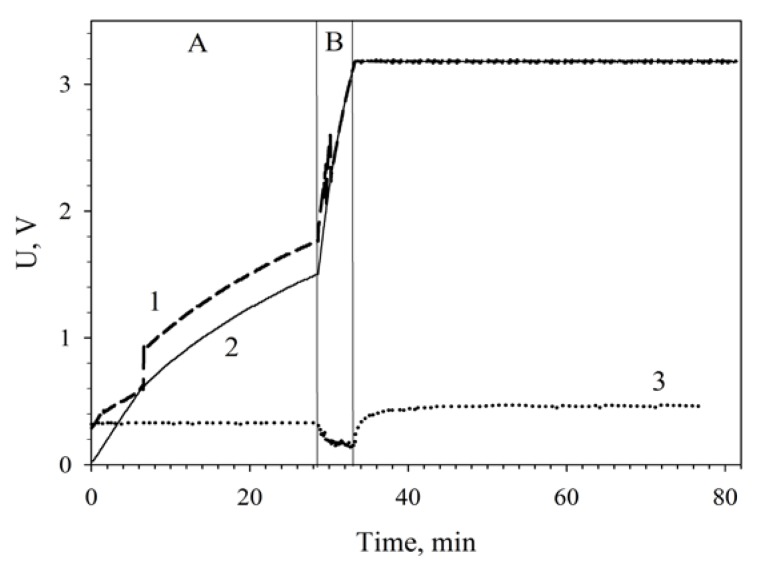
Charging a 6800-μF capacitor from series-connected MWCNT-modified MFCs using a converter unit: 1, charge voltage; 2, voltage across the accumulating capacitance; 3, input voltage coming to the converter. (A), low-efficiency slow charge phase; (B), high-efficiency fast charge phase. Registration of the capacitor total charge after a 48-h continuous operation of the MFCs is shown.

Figure 7 plots the charging of a 6800-μF capacitor by two MWCNT-modified MFCs connected in series. The low-efficiency slow charge phase (up to 1.7 V) was 28 min; the high-efficiency fast charge phase, 4 min; the total capacitor charge time was 32 min.

**Figure 8:**
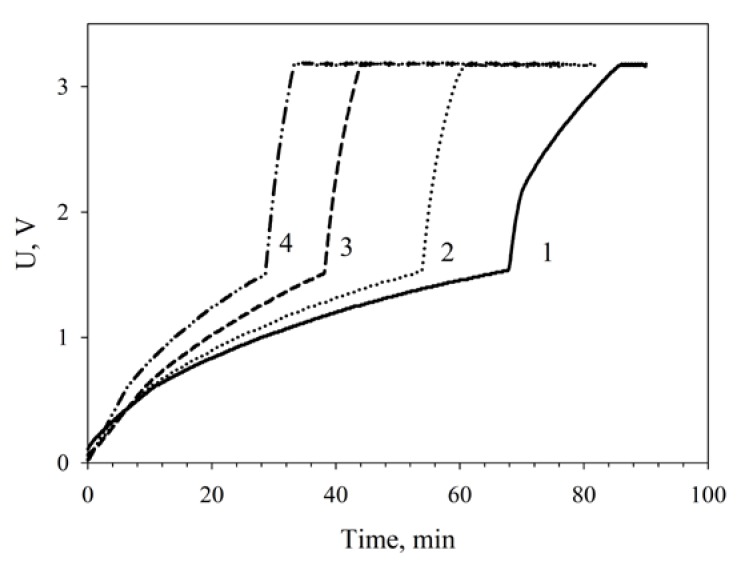
Dependence of the charging of a 6800-μF capacitor on MFC operating conditions: 1, nonmodified MFCs; 2, MWCNT-modified MFCs; 3, MWCNT-modified MFCs after a 24-h operation; 4, MWCNT-modified MFCs after a 48-h operation.

The dependence of capacitor charge time on MWCNT-modified MFC functioning time as compared with nonmodified MFCs is given on Figure 8. Two nonmodified microbial fuel cells charged a capacitor of 6800 μF in 80 min; therefore, the larger part of the time (60 min) was the slow charge phase. At a modification of the MFC by nanotubes the total charge time decreased to 60 min, the slow charge time being 52 min. The time occupied by the slow charge phase decreased by 23%; the time of the fast charge phase, by 34%.

It should be noted that two series-connected nanotubes-modified MFCs efficiently charged a capacitor of 6800 μF in 42 min after a 24-h continuous operation. After a 48-h continuous operation of the MFCs, the capacitor charge time decreased more than two times as compared with two nonmodified cells to make 30 min. It can be assumed that this improvement of MFC parameters is due to the phenomenon of adaptation presented, e.g., in references [43,44].

The charged capacitor contained a charge of 21 × 10^−3^ C, having accumulated energy of 32.7 mJ, which made it possible to maintain the luminescence of a light-emitting diode (L-1154SURDK, Kingbright; 2.0 V, 20 mA) or to rotate an electric motor rotor (M25E-4L, MITSUMI; 3.0 V, 100 mA) in a short-time regime.

Accumulated charge was used to power a low-energy amperometric biosensor based on a Clark-type electrode. The value of consumed current for an ~0.3 mm-diameter platinum electrode was of the order of 10^–9^ А. A charged capacitor of 6800 μF provided the operation of a biosensor based on a Clark-type oxygen electrode for 27 h (biosensor connections and operation scheme not shown).

The charged capacitor of 30,000 μF (charging time, 80 min) was used to power a device with a Bluetooth transmitter and three sensors (for temperature, humidity, light intensity) for 2 min net time (preliminary data presented at the IEEE 4th World Forum on the Internet of Things [45]). Such devices can be used as part of the Internet of Things technology to monitor various parameters of the environment with sensors’ polling period of approximately once per 2 h.

## 4. Conclusions

In the course of the research, we investigated the effects of polymers for immobilizing bacterial cells on the anode surface of a microbial fuel cell on its bioelectrochemical characteristics. An MFC with chitosan gel used for immobilization was shown to have the lowest internal resistance (1111 Ohm cm^2^) and to develop the maximal power (7.6 μW/cm^2^). Additional introduction of carbon nanotubes into chitosan gel enabled decreasing the MFC internal resistance to 977 Ohm cm^2^ and increasing the developed power to 10.6 μW/cm^2^. Modification of PVA and mPVA polymers by carbon nanotubes also reduced the MFC internal resistance by 2.5 and 12.2% and increased the developed MFC power by 20.7 and 18.5%, respectively. Thus, a promising polymer for immobilization of bacterial cells on the graphite anode surface is chitosan modified by carbon nanotubes. Addition of nanotubes leads to an increase in the MFC power due both to the increased biocatalyst/electrode-surface contact area and to the facilitated charge transfer along the formed conductive network of nanotubes on the surface of the electrode. Microbial fuel cells are a new and promising technology, and its cost-effectiveness is still being estimated. There are some reports [46] that inexpensive materials are capable of achieving more cost-effective energy generation than high-performing materials despite generating lower power. In this work low-cost materials such as graphite rods and chitosan were used. Bacterial cells are also more cost-effective than purified enzymes for energy generation. In this work we present a solution which allows MFCs to power several IoT devices due to the accumulation of energy via a boost converter unit, which increased MFC voltage from 0.3 V to a value of 3.2 V. We are hoping that this solution could be useful for such applications as waste water management and sewage analysis. The obtained results form the basis for application of microbial fuel cells in smart-home and smart-city technologies.

## Figures and Tables

**Table 1:** Values of the bioelectrochemical characteristics of electrodes modified by various polymers.

	*R*_ct_, Ohm cm^2^	*R*_in_, Ohm cm^2^	*P*_sp_, μW/cm^2^	∆*I*, μА
PVA	1255 ± 213; 16.9%	1251 ± 201; 16.1%	6.56 ± 0.74; 11.3%	134.7 ± 9.0; 6.7%
mPVA	1294 ± 103; 8.0%	1455 ± 192; 13.2%	6.10 ± 0.32; 5.3%	139.2 ± 7.5; 5.4%
Chitosan	1069 ± 59; 5.5%	1111 ± 71; 6.4%	7.63 ± 0.28; 3.4%	159.0 ± 5.9; 3.7%

Note: mean values of 10 measurements, standard deviations from the mean values and the measuring errors in per cent are given.

**Table 2:** Values of the bioelectrochemical characteristics of electrodes modified by various polymers and multiwalled carbon nanotubes (MWCNT).

	*R*_ct_, Ohm cm^2^	*R*_in_, Ohm cm^2^	*P*_sp_, μW/cm^2^	∆*I*, μА
PVA	908 ± 52; 5.7%	1219 ± 43; 3.7%	7.92 ± 0.51; 6.4%	139.6 ± 4.8; 3.4%
mPVA	1088 ± 70; 6.5%	1277 ± 54; 4.2%	7.23 ± 0.80; 11.0%	147.2 ± 10.0; 6.8%
Chitosan	722 ± 30; 4.2%	977 ± 57; 5.9%	10.56 ± 0.78; 7.8%	194.0 ± 14.4; 7.4%

Note: mean values by 10 measurements, standard deviations from the mean values and the measuring errors in per cent are given.

**Table 3:** MFC characteristics and capacitor charge times.

Cell	Power, μW	Internal Resistance, Ohm	Charging Time of 6800 μF Capacitor
Single MFC, nonmodified	80	200	Does not charge
Single MFC, MWCNT-modified	110	160	Does not charge
Series-connected nonmodified MFCs	140	380	1st charge cycle, 80 min; 2nd charge cycle, 70 min
Series-connected MWCNT-modified MFCs	198	290	1st cycle, 60 min; after 48 h, 32 min
